# Initial assessment of the nutritional quality of the space food system over three years of ambient storage

**DOI:** 10.1038/s41526-017-0022-z

**Published:** 2017-06-09

**Authors:** Maya Cooper, Michele Perchonok, Grace L. Douglas

**Affiliations:** 1 Leidos, Inc., Houston, TX 77258 USA; 2 Human Health and Performance Directorate, NASA Johnson Space Center, Houston, TX 77058 USA

## Abstract

Processed and prepackaged space food is the main source of nutrition for crew aboard the International Space Station, and likely will continue to be the main source of nutrition for future exploration missions. However, very little information is available on the nutritional stability of space foods. To better understand their nutritional stability, 24 micronutrients were measured in 109 space foods stored over 3 years at room temperature. Our analysis indicated that potassium, calcium, vitamin D, and vitamin K concentrations in the food may not be adequate to meet the recommended daily intake requirements even before storage. Decreases in vitamins A, C, B_1_, and B_6_ were observed during storage. Notably, vitamins B_1_ and C may degrade to inadequate levels after 1 year and 3 years, respectively. This assessment suggests that different technological approaches will be required to stabilize processed foods to enable spaceflight missions over 1 year.

## Introduction

Nutritional intake has significant consequences for the health and performance of astronauts during and after space flight. The primary focus in nutritional research has been on bioavailability and nutritional requirement modifications for crewmembers,^1^ and little information has been available regarding the stability of nutrients in the shelf stable food system. A processed and prepackaged food system is the main source of nutrition for current crews and exploration crews will likely use the same system. Logistics and resource availability prevent the implementation of fresh or refrigerated food systems,^2^ and supplements cannot replicate the numerous bioactive compounds and the synergies that are provided through whole foods and a balanced diet.^3^ Crewed space missions beyond low-Earth orbit require a system that can deliver high quality food provisions, ideally without conditioned storage, for up to 5 years. At the time of consumption, the food must be acceptable in quality and must deliver the recommended intake of macronutrients, vitamins, and minerals.

The current International Space Station (ISS) food system is composed of processed foods that are vacuum-packaged in high-barrier laminates with an aluminum foil layer, microbiologically safe, and compatible with microgravity.^2^ The freeze-drying, irradiation, or retort thermostabilization processing employed to achieve food safety induce chemical and structural changes that alter the food quality and stability.^4^ Processing and storage impact most vitamins,^5^ but degradation is specific to the food matrix, processing history, packaging, and shelf life parameters. Available stability data is not specific to space food parameters and cannot be accurately extrapolated.

Only five foods from the spaceflight food system have previously been evaluated for multi-year nutritional stability, and the results indicate that vitamins B_1_, B_9_, K, and C significantly degrade on a food-specific basis.^6^ The nutritional adequacy of the 203 current space food items has only been evaluated through database estimates that do not account for spaceflight-specific parameters or extended storage, but the estimates inform the standard spaceflight food menu composition. By analyzing representative examples from the spaceflight food system, this study provides an initial assessment of the nutritional adequacy of the current space food system. The stability results provide a baseline for comparison with future processing and storage technologies, which will enable advancement towards a system with adequate nutritional stability on future exploration missions.

## Results

**Fig. 1:**
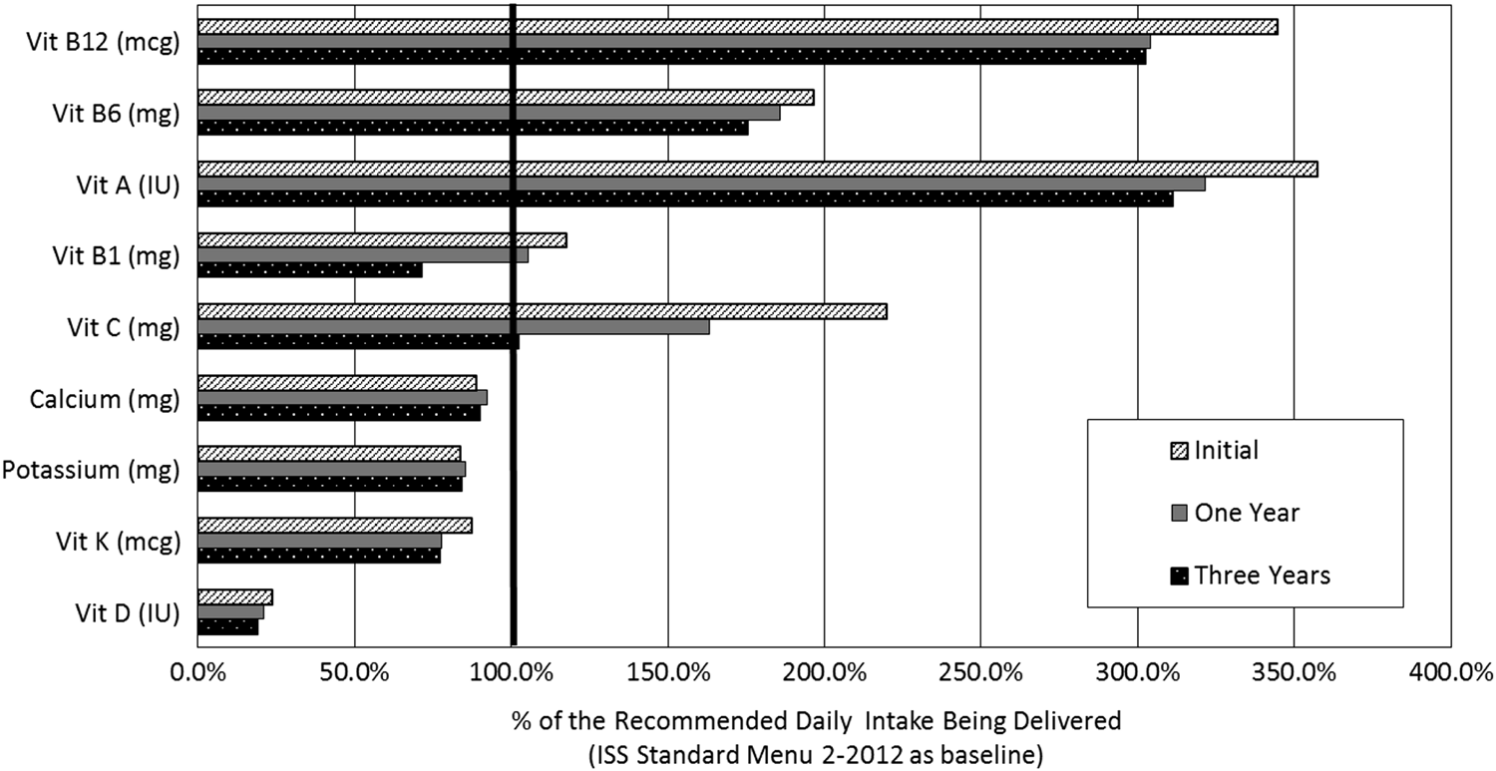
Expected vitamin delivery for aging space diet consumed according to the ISS Standard Menu. Total concentrations of vitamins D and K as well as calcium and potassium are below recommended intakes post-processing. Vitamin C and B_1_ may degrade to inadequate concentrations within 3 years of 21 °C storage; vitamins A, B_6_, and B_12_ decline but sufficient concentrations remain after 3 years. Beverages were excluded from this analysis because they are not currently part of the standard menu, and are only selected based on crew preference

Post-production analysis indicated that vitamin D, vitamin K, potassium, and calcium were not adequate to meet recommended nutrient daily intake, assuming dietary compliance to the standard spaceflight food menu (Fig. 1). Vitamin D is generally low in food. The deficit of vitamin D, largely due to lack of exposure to sunlight, has been mitigated with a supplement on ISS. Both potassium and calcium concentrations were approximately 20% lower than recommended intake levels and vitamin K had a projected 13% daily shortfall.

The more labile vitamins in spaceflight food degraded during 21 °C temperature storage over a 3-year storage period (Fig. 1). Vitamins B_1_ and C declined rapidly, but several vitamins (including A, B_6_, and B_12_) only showed minor degradation or were available in enough foods that nutrient delivery remained adequate.

**Fig. 2:**
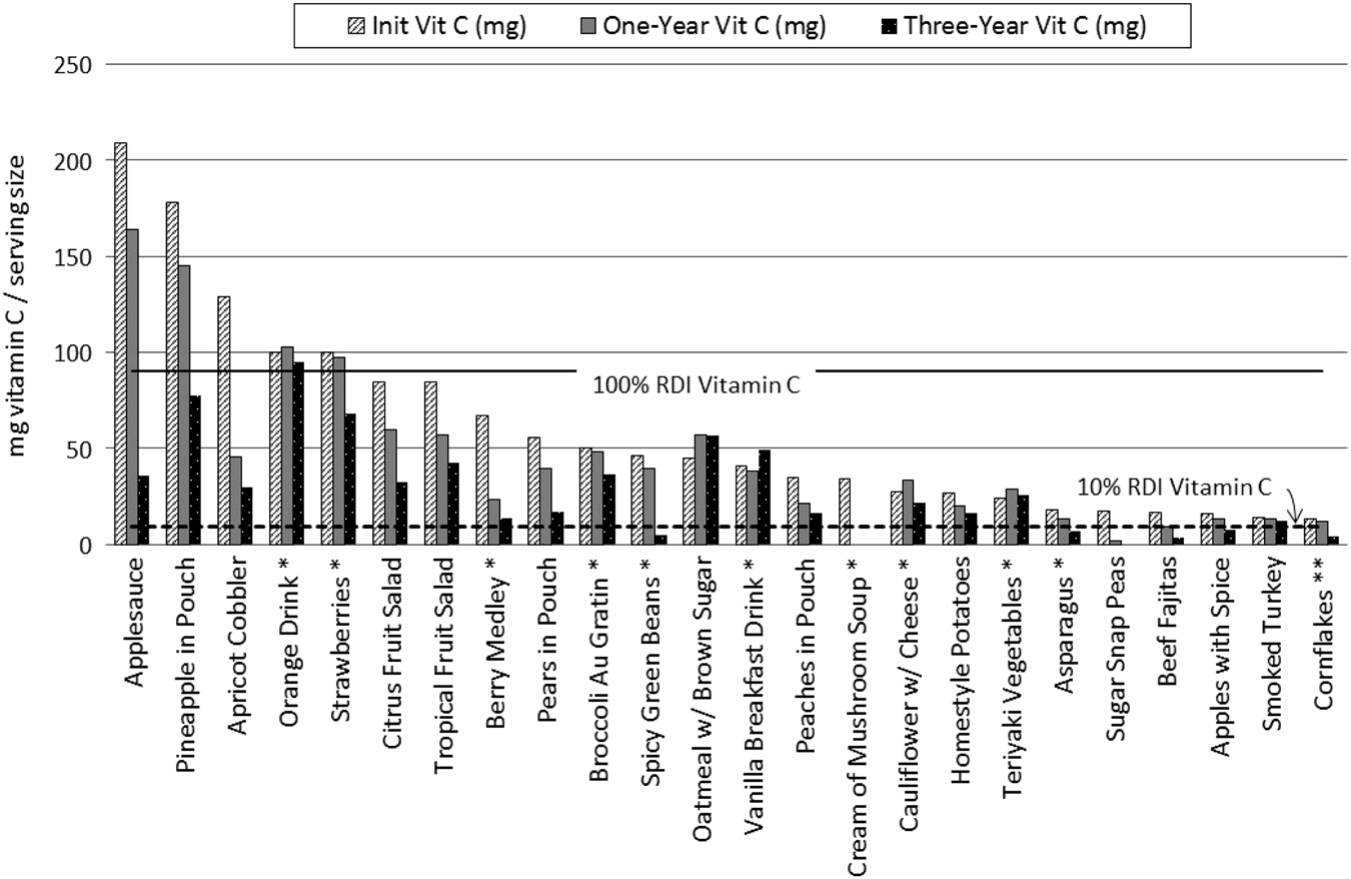
Vitamin C concentrations in space foods over 3 years of 21 °C storage generally declined from the initial concentration. The rate of decline was most dependent upon the primary food matrix and formulation. Note: Increases in content from the initial concentration in fortified products is likely due to encapsulation, which can impede complete chemical detection until the encapsulation itself degrades over time

The nutritional stability trends did not support a difference between processing methods after 3 years for most vitamins. However, the stability of several vitamins varied with food formulation or matrix. The vitamin C content in most fruit products degraded between 32 and 83% after 3 years of storage, with no attributable differences to ascorbic acid fortification status (Fig. 2). Vitamin C appeared to be more stable in freeze-dried products that offered protection against oxidation (products with sauces) and in powdered fortified beverages.

Vitamin B_6_ also varied in stability, degrading an average of 14.5% in the foods with the highest concentration over 3 years of ambient storage. Chicken products and beef products had slightly higher B_6_ degradation averages at 26 and 22%, respectively.

**Fig. 3:**
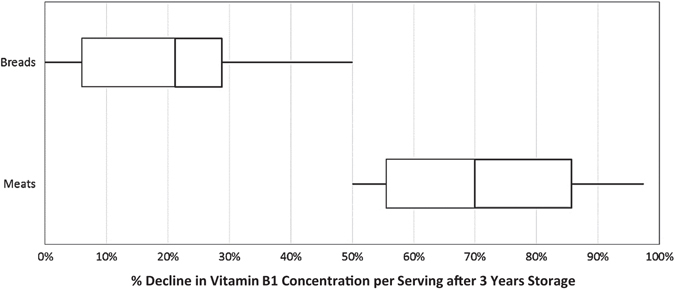
Comparison of vitamin B_1_ stability in breads and meats after 3 years of 21 °C storage. Vitamin B_1_ concentrations in breads were sustained at higher levels than B_1_ concentrations in meats. Multiple factors, including processing, food matrices, and fortification may have contributed to the difference in stability over time

Thiamin was more stable in bread products than in animal products (Fig. 3). Several potential factors, such as degradation due to irradiation and thermal processing, the individual food matrices, or the use of thiamin mononitrate—a highly stable form of B_1_—in bread products, may have led to the variance in thiamin stability.

## Discussion

While based on a single set of analyses, our study indicates that the current space food system may not provide adequate vitamin C and B_1_ for 3-year exploration missions. Other vitamins, such as B_9_ and B_12_, may also have stability issues but require further analysis. Some vitamins degraded on a more food-specific basis (i.e., vitamins B_2_ and E), but did not substantially shift the nutritional delivery of the space food system (data not shown). Many vitamins are available in enough foods and at adequate concentration so that they are predicted to be available for up to 5 years with compliance to the standard menu. Nonetheless, the necessary nutrients could still be insufficient if crewmembers do not adequately balance their diets or intake the nutrient-stable foods. Given the limited food supply during missions, the food choices of one crewmember will affect the food choices remaining for other crewmembers.

Food and nutrient intake by the crew is a complex issue. This study addresses a fraction of that complexity, highlighting potential nutrient delivery concerns from a multi-year stored food system, which may have implications for future exploration crews. Although fortification or supplementation could be suggested as a nutritional countermeasure, capsule supplements may similarly degrade and would require compliance. Additionally, synthetic vitamin delivery would neglect the bioactive compounds provided through whole and processed foods, and the benefits that these compounds might provide in a chemically stable context. A highly acceptable and nutritious food system best ensures caloric and nutrient delivery to the crew, and efforts to stabilize or develop whole foods for this system, through alternative processing or reduced temperature storage, are recommended.

Current research is investigating the stability of foods processed with microwave-assisted thermal stabilization (MATS) compared to current thermal processing methods, and conjointly, investigating food stability after extended frozen or refrigerated storage for all space foods. A shorter thermal exposure in MATS processing and the slowed kinetic rates of chemical degradation at reduced temperatures is expected to improve vitamin stability and increase shelf life.^7^ Furthermore, a bioregenerative salad crop system is being explored in ground chambers and on the ISS to determine reliable infrastructure and horticulture methods that will enable crops grown in spaceflight to supplement nutrition for the crew.^8^ As infrastructure development, crop selection, and system reliability are advanced and validated, it is expected that bioregenerative food crops will become an important source of nutrition to crew.

## Methods

One-hundred-nine of 203 foods currently available on the ISS standard menu were selected for nutritional investigation based on method of stabilization and the primary food matrix. The foods were processed and packaged according to the current spaceflight specifications and then stored at 21 °C for up to 3 years.

Three packages of each food item were immediately sent after production to a reference laboratory (Covance, Madison, WI, USA) for composite analysis of 24 vitamins and minerals following the Official Methods of Analysis of AOAC International.^9, 10^ Samples were similarly sent after 1 and 3 years of storage in a 21 °C incubator (temperature on ISS). Foods were prepared accordingly to ISS instructions (rehydration) prior to analysis. The data was categorized by food group and used to qualitatively indicate nutritional degradation of the food system based on the standard spaceflight menu.

### Data availability

Data are available from the NASA Life Sciences Data Archive: https://lsda.jsc.nasa.gov/lsah_home1.aspx.
